# Corticostriatal Dysfunction in Huntington’s Disease: The Basics

**DOI:** 10.3389/fnhum.2016.00317

**Published:** 2016-06-28

**Authors:** Kendra D. Bunner, George V. Rebec

**Affiliations:** Department of Psychological and Brain Sciences, Program in Neuroscience, Indiana UniversityBloomington, IN, USA

**Keywords:** glutamate, dopamine, cortiostriatal circuitry, electrophysiology, Huntington’s disease

## Abstract

The main input to the basal ganglia, the corticostriatal pathway, shows some of the earliest signs of neuropathology in Huntington’s disease (HD), an inherited neurodegenerative condition that typically strikes in mid-life with progressively deteriorating cognitive, emotional, and motor symptoms. Although an effective treatment remains elusive, research on transgenic animal models has implicated dysregulation of glutamate (Glu), the excitatory amino acid released by corticostriatal neurons, in HD onset. Abnormalities in the control of Glu transmission at the level of postsynaptic receptors and Glu transport proteins play a critical role in the loss of information flow through downstream circuits that set the stage for the HD behavioral phenotype. Parallel but less-well characterized changes in dopamine (DA), a key modulator of Glu activation, ensure further deficits in neuronal communication throughout the basal ganglia. Continued analysis of corticostriatal Glu transmission and its modulation by DA, including analysis at the neurobehavioral level in transgenic models, is likely to be an effective strategy in the pursuit of HD therapeutics.

## Introduction

Aberrant function of basal ganglia circuitry plays an important role in multiple neuropathological conditions (Reiner, 2010). Here we focus on Huntington’s disease (HD), a dominantly inherited and ultimately fatal neurodegenerative disorder characterized by near-total loss of cognitive, emotional, and motor control (Cepeda et al., 2007; Zuccato et al., 2010). Early signs of HD neuropathology emerge in the striatum, the main input structure of the basal ganglia, and the cerebral cortex, which supplies the striatum with the associative, limbic, and motor information necessary to select and guide appropriate behavioral responses despite widely varying circumstances and contexts (Kreitzer and Malenka, 2008; Milnerwood and Raymond, 2010). In effect, the corticostriatal system supports the production of skilled yet flexible behavioral actions that define a healthy life. HD disrupts this system by interfering with the mechanisms by which cortical and striatal neurons communicate.

## Symptoms and Neuropathology of Huntington’s Disease

HD afflicts approximately 1 in 10,000 persons of European descent. Other populations are also affected but at a slightly lower incidence (Harper, 2001; Rawlins et al., 2016). The largest and best characterized population with HD is localized to the region of lake Maracaibo, Venezuela. In fact, it was with this population the underlying genetic cause was discovered (Gusella et al., 1983).

HD is caused by an expansion of the polyglutamine (CAG) repeat in the *huntingtin* (*htt*) gene located on exon 1 of chromosome 4 (The Huntington’s Disease Collaborative Research Group, 1993), which encodes the protein huntingtin (HTT). In healthy individuals, *htt* alleles contain 6–30 CAG repeats; more than 30 repeats and the gene is considered mutated. An expansion of 36 or more CAG repeats will result in disease onset with full penetrance occurring at 40 or more repeats (Rubinsztein et al., 1996). An intermediate number of repeats (e.g., 30–36) is associated with germline instability and can lead to *de novo* HD, indicating a higher prevalence of HD in general populations with longer CAG repeats (Bates et al., 2015). Although there is considerable variability, the onset and severity of symptoms is inversely correlated with the number of CAG repeats; the greater the CAG expansion, the earlier the onset of symptoms (Nørremølle et al., 1993; Harper and Jones, 2002; Kumar et al., 2010). Symptoms typically begin to manifest around midlife, 35–45 years of age, with the majority of patients having between 40 and 50 CAG repeats. An expansion greater than 50 CAG repeats results in juvenile onset (Telenius et al., 1993).

Following clinical diagnosis, usually triggered by motor symptom onset, HD is fatal within 15–20 years. During the initial stages of the disorder, HD patients experience cognitive deficits in mood, attention, procedural memory and executive functions (Lemiere et al., 2004; Estrada-Sánchez et al., 2015a). They also experience mild motor abnormalities such as tremor or tics. Following these mild changes, a hyperkinetic stage emerges. Motor abnormalities, primarly chorea, the defining feature of HD, manifests with abrupt and uncontrolled exaggeration of gestures and spontaneous movements of the trunk, face, and limbs. These movements cannot be volunatrly suppressed and worsen during periods of heighted stress (Kremer, 2001). Motor skills continue to deteriorate affecting gait, speech and swallowing. During this time patients also suffer dramatic weight loss and muscle wasting despite maintaining a high caloric diet (Estrada-Sánchez et al., 2015a). Eventually choreic movements are replaced by bradykinesia and rigidity and ultimately results in death primarily due to pneumonia and heart disease (Estrada-Sánchez and Rebec, 2012).

Neuropathological features of HD include: reduced brain weight, enlarged lateral ventricles, and decreased striatal and cortical volume. The gross atrophy of the caudate-putamen (striatum) results mainly from loss of medium spiny neurons (MSNs), which account for >90% of the striatal neuronal population. Striatal neuronal loss is usually accompanied by loss of cortical pyramidal neurons (CPNs), primarily in motor and premotor areas (Aylward et al., 1998; Rosas et al., 2001; Menalled et al., 2002). Because CPN targets include MSNs, the degeneration and loss of these neurons indicate impaired corticostriatal connectivity and dysfunctional information processing (Hamilton et al., 2003; Miller et al., 2011; Estrada-Sánchez and Rebec, 2012). Thus, although mutant HTT is widely expressed in brain and body, primary neuropathology occurs in the cerebral cortex and the striatum. This review will focus on changes in corticostriatal circuitry and how glutamate (Glu), the transmitter released by CPNs, and its interaction with dopamine (DA), a key modulator of striatal function, affect this circuitry in HD. Multiple animal models of HD developed soon after identification of the *htt* gene form the basis for this line of research.

## Animal Models

A few years following identification of the gene, transgenic rodent models were developed in order to investigate mechanisms underlying the HD phenotype. Genetic murine models can be separated into three groups: transgenic truncated models, transgenic full-length models, or knock-in (KI) models. Truncated models solely express the first exon of the *htt* gene, where the CAG expansion occurs. Full-length models express the entire human mutant *htt* gene. Both of these transgenic models contain two non-mutated endogenous *htt* alleles making the mutant HTT to HTT ratio differ from that seen in humans. This raises translational concerns as some cellular alteration in the truncated and full-length models may not be an accurate reflection of the human condition. KI models avoid this translational confound by directly inserting the CAG expansion into exon 1 of the endogenous *htt* gene; thereby mirroring the genetic construct of HD patients.

### Truncated Models

The truncated R6 line is the oldest and one of the most studied transgenic mouse models. R6/2 mice, with a CAG repeat length of ~150, have an aggressive phenotype. R6/2 mice have a shortened lifespan of 3–5 months with symptom onset beginning as early as 1 month of age (Mangiarini et al., 1996). R6/1 mice have a longer life expectancy than R6/2 mice. With 110 to 115 CAG repeats, R6/1 mice become symptomatic between 4 to 5 months with more robust motor symptoms emerging around 6 to 7 months of age followed by death at 10 to 14 months (Mangiarini et al., 1996; Naver et al., 2003).

The R6 line has neuronal intracellular and intranuclear aggregates contacting the mutant HTT fragment (Davies et al., 1997); as well as reduced striatal volume (Stack et al., 2005). Motor deficits including tremor, reduced climbing, and clasping has been observed in R6 truncated models (Mangiarini et al., 1996; Miller et al., 2008). Susceptibility to epileptic seizures (Mangiarini et al., 1996) and decreased learning has also been observed in these truncated models (Murphy et al., 2000). These behavioral changes are correlated to neurochemical changes in basal ganglia circuitry (Bibb et al., 2000; Ariano et al., 2002; Johnson et al., 2006; Miller et al., 2008; Ortiz et al., 2010).

### Full-Length Models

Two full-length transgenic HD models have been developed: the yeast artificial chromosome (YAC) and bacteria artificial chromosome (BAC). Several lines of the YAC mouse model have been generated based on the number of CAG repeats: YAC18 (control), YAC46, YAC72, and YAC128. Compared to truncated models, YAC and BAC mice have a slower disease progression. As is the case with humans, the YAC128 mouse model displays striatal followed by cortical atrophy as well as hyperkinetic followed by hypokinetic activity in an age-dependent manner (Slow et al., 2003; Van Raamsdonk et al., 2005). The BAC model, which carries 97 CAA-CAG repeats, also exhibits a progression motor deficits, decreased striatal and cortical volume, and decreased MSN synaptic activity (Gray et al., 2008). BACHD mice have also shown abnormal striatal and cortical firing patterns (Estrada-Sánchez et al., 2015b).

The YAC128 mouse model is one of the few transgenic models exhibiting both the hyper- and hypoactive phenotype seen in humans as well as striatal followed by cortical atrophy (Slow et al., 2003; Van Raamsdonk et al., 2005). In the YAC128 mouse model, by 3 months of age a hyperkinetic phenotype emerges this is then followed by progressive motor deficits. By 9 months of age, motor deficits are apparent and the hypokinetic phenotype is accompanied by striatal atrophy; when the animal reaches 12 months of age cortical atrophy also starts to occur (Slow et al., 2003). Because the onset of symptoms does not occur as rapidly in the YAC128 model as truncated models; initial symptoms seen in humans as well as neurochemical changes throughout the progression of this biphasic (hyper- and hypo-) active disorder can be studied in more depth.

### Knock-in Models

Multiple HD KI models have been created. KI models carry the expanded CAG repeats within the native murine *htt* gene, closely mimicking the genetic context of patients with HD. KI models provide stronger construct validity compared to other transgenic rodent models. However the protracted phenotype observed in KI models has limited their use. Current KI mouse models show subtle behavioral, histopathological, and molecular phenotypes compared to the transgenic models that over express mutant HTT (truncated and full-length models; Chang et al., 2015). The CAG140 KI mouse model has a normal lifespan, with overt symptoms emerging after 20 months of age (Hickey et al., 2008). CAG140 mice do have the characteristic nuclear aggregates and decreased striatal volume seen in other models (Menalled et al., 2003; Hickey et al., 2008). The Q175 KI model was derived by a spontaneous germline CAG expansion from the previously constructed CAG140 KI mouse model (Menalled et al., 2012). Initial characterization of this new mouse model showed decreased body weight, body tremor, abnormal gait, and activity level. Decreased striatal and cortical volume has also been observed along with decreased cognitive ability (Heikkinen et al., 2012; Menalled et al., 2012). These behavioral changes observed in HD KI models mirror changes seen other mouse models and HD patients (Mangiarini et al., 1996; Slow et al., 2003; Miller et al., 2011; Estrada-Sánchez et al., 2015a).

## Corticostriatal Pathway Overview

As the primary input nucleus of the basal ganglia, the striatum receives excitatory afferents from the entire cortical mantel as well as the thalamus (Kreitzer and Malenka, 2008). Because the primary neuropathology of HD involves the loss of MSNs and CPNs, the HD behavioral phenotype is likely due to dysfunction of the cortical-basal ganglia system. Therefore a greater understanding of the corticostriatal pathway and its activity is necessary for a fuller understanding of both HD pathology and behavioral phenotype.

### Cortex

In humans, the cortical mantel is divided into six layers with astrocytes homogenously distributed throughout. The different types of cortical neurons make local cortical connections between cells of different layers as well as send and receive projections from other brain regions, such as the striatum. Layer III, V, and VI all contain CPNs. CPNs from layer III and V are the primary input to the basal ganglia, brain stem, and spinal cord. CPNs from layer VI send projection to the thalamus (McGeorge and Faull, 1989; Shipp, 2007; Estrada-Sánchez and Rebec, 2013). Post-mortem tissue taken from HD patients has a 30% reduction in CPNs for these three cortical layers (Cudkowicz and Kowall, 1990; Hedreen et al., 1991; Sotrel et al., 1991; Heinsen et al., 1994). While the entire cortical mantel projects to the striatum, this review will be referring to CPNs of the motor cortex due to the motor cortex’s involvement in execution and control of voluntary movements. This reduction of CPNs as well as studies showing dysfunction CPNs neuronal firing (Walker et al., 2008) potentially underlies the HD behavioral phenotype.

Two types of striatal-projecting CPNs have been identified: (1) pyramidal tract (PT)-type neurons; and (2) intra-telencephalically projecting (IT)-type neurons. PT-type neurons project to ipsilateral striatum, thalamus, and subthalamic nucleus (STN) as well as the regions of the spinal cord and brain stem. IT-type neurons project to the ipsilateral and contralateral striatum and other cortical layers (Shepherd, 2013). PT-type neurons are mainly found in the lower cortical layer V and IT-type neurons are primarily located in layer III and upper half of layer V. The different morphological features and location of PT- and IT-type neurons suggest these two types of neurons differentially target striatal MSNs, with IT-type neurons differentially targeting MSN of the direct pathway and PT-type neurons preferentially innervate MSNs of the indirect pathway (Reiner et al., 2003; Parent and Parent, 2006; Chen et al., 2013).

### Striatum

MSNs are GABAergic neurons that constitute 95% of the neuronal population of the striatum. Interneurons in the striatum are also primarily GABAergic, with some cholinergic interneurons (Kreitzer and Malenka, 2008). There are two major subtypes of striatal MSNs based on their protein expression and their axonal projections: (1) Striatonigral MSNs express substance P (subst P), dynorphin, and D1-like DA receptors. They project directly to the basal ganglia output nuclei: internal globus pallidus (GPi) and substantia nigra pars reticulata (SNr); and (2) Striatopallidal MSNs contain enkephalin (enk) and express D2-like DA receptors. Striatopallidal MSNs projects to the external globus pallidus (GPe) and are part of the indirect pathway. Both striatonigral and striatopallidal MSNs integrate glutamatergic cortical and thalamic inputs and relay that information to downstream basal ganglia nuclei (Kreitzer and Malenka, 2008).

**Figure 1:**
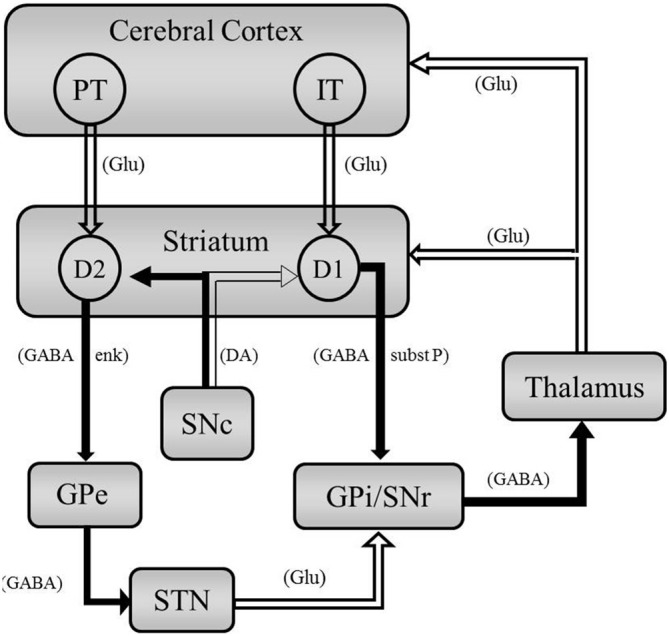
**Simplified schematic representation of basal ganglia circuitry highlighting cortical and striatal outputs.** Open arrows represent excitation, closed arrows represent inhibition. Neurotransmitters are indicated in parenthesis. Intra-telencephalically projecting (IT)-type CPNs project to the striatongiral D1-enriched MSNs of the direct pathway. Pyramidal tract (PT)-type CPNs project to the stratopallidal D2-enriched MSNs of the indirect pathway. Abbreviations: Glu, glutamate; enk, enkephalin; subst P, substance P.

These two subtypes of MSNs have been used to segregate basal ganglia circuitry into two pathways: the direct pathway containing striatonigral D1-enriched MSNs and the indirect pathway containing striatopallidal D2-enriched MSNs. These pathways are believed to act in opposing ways to control movement. According to this view, the direct pathway, which consists of the striatonigral MSNs, initiates movement; whereas, the indirect pathway, consisting of striatopallidal MSNs, inhibits movement (Alexander and Crutcher, 1990). While these pathways are not completely isolated (Haber et al., 2000; Cui et al., 2013), for simplicity, we will discuss them as parallel circuits, depicted schematically in Figure 1.

The direct pathway is thought to facilitate movement (Albin et al., 1989; DeLong, 1990). Striatonigral MSNs in the striatum receive excitatory, glutamatergic projections from the cortex and thalamus. This results in excitation of the striatal GABAergic MSNs, which in turn inhibits the GABAergic projection in the GPi/SNr. The inhibition of the inhibitory GABAergic neurons in the GPi/SNr results in less inhibition on the thalamus glutamatergic neurons. This results in excitation of the motor cortex and initiation of voluntary movement. By this schematic, dysregulation of striatonigral MSN projection neurons would result in rigidity and bradykinesias.

The indirect pathway is presumed to inhibit movement (Albin et al., 1989; DeLong, 1990; Durieus et al., 2009; Kravitz et al., 2010). Striatopallidal MSNs inhibit GABAergic neurons in the GPe. This in turn results in less inhibition on the glutamatergic projection in the STN. The glutamatergic projections from the STN excite the GABAergic neurons in the GPi/SNr which results in greater inhibition to the thalamus and decreased signaling to the motor cortex (Alexander and Crutcher, 1990). Thus dysregulation of MSNs in the indirect pathway result in uncontrollable voluntary movements, such as chorea and tremor (Bateup et al., 2010).

Glu projections from the cortex are not the only influence on basal ganglia circuitry. DA projections from the substantia nigra pars compacta (SNc) play a modulatory role. Evidence suggests D1-like receptor signaling facilitates glutamatergic signaling in direct pathway, striatonigral MSNs. In contrast, D2-like receptor activation inhibits MSNs in the indirect pathway (Figure 1; Surmeier et al., 2007). Therefore DA projections from the SNc results in excitation (D1-like) or inhibition (D2-like) of the direct or indirect pathway, respectively, the ultimate results is facilitation of movement. Therefore a dysregulation of Glu or DA, which modify the excitatory responses induced by Glu (Kiyatkin and Rebec, 1999; Cepeda and Levine, 2006), could results in uncontrolled involuntary movements or bradykinesia.

## Dysregulation of the Corticostriatal Pathway

Motor function is in part shaped by CPNs projecting to striatal MSNs in the so-called corticostriatal pathway. The cerebral cortex and striatum are the most affected brain areas in HD, with massive MSN loss in the striatum. Not all MSNs however are equally vulnerable (Vonsattel and DiFiglia, 1998; Galvan et al., 2012). Morphological evidence shows time-dependent, differential alterations in the two populations of MSNs. MSN in the indirect pathway tend to be preferentially lost prior to MSN in the direct pathway. Evaluation of indirect striatopallidal MSNs and their projections (i.e., enk) are lost in postmortem tissue of symptomatic patients and in presymptomatic and early symptomatic brains of HD mouse models. In contrast, direct striatonigral MSNs projecting subst P are not as greatly affected and in some cases appear unaffected till later, advance stages of the disease (Albin et al., 1992; Sapp et al., 1995; Menalled et al., 2000).

In keeping with the direct/indirect model of basal ganglia movement control, the preferential loss of MSN in the indirect pathway can result in the inability to control voluntary movement, resulting in a hyperkinetic phenotype (chorea). The dysfunction of the direct pathway in the later stage of the disease would result in an inability to facilitate movement; resulting in a rigidity and bradykinesia (see above). Thus changes in output from striatal MSNs, which receives Glu input from the cortex, correlates with the behavioral phenotype of HD.

Particular focus has been placed on the neuronal activity of the cortex and striatum. Regardless of genetic construct and phenotypic onset, changes ranging from membrane properties to impaired neuronal processing in the corticostriatal pathway have been identified in multiple mouse models of HD (Walker et al., 2008; Miller et al., 2011; Heikkinen et al., 2012; Estrada-Sánchez et al., 2015a,b). Abnormal MSN activity has been observed in presymptomatic and symptomatic transgenic mouse models (Rebec et al., 2006; Miller et al., 2008, 2011; Cayzac et al., 2011; Estrada-Sánchez et al., 2015b). Abnormal CPN activity has also been observed in HD mouse models (Walker et al., 2008, 2011). Multiple transgenic mouse models show increased firing rate with decreased burst firing and firing variability in the dorsal striatum and primary motor cortex. A decrease in synchronous firing and coherent bursting was also observed in the cortex and striatum of transgenic murine models (Walker et al., 2008; Höhn et al., 2011; Miller et al., 2011; Estrada-Sánchez et al., 2015b). Changes in neuronal activity level and variability along with changes in burst firing, which is involved in synaptic plasticity and enhanced information transmission, demonstrate neuronal dysfunction in the striatum and cortex thereby indicating a communication problem between the cortex and dorsal striatum.

*In vitro* electrophysiology analysis showed resting membrane potential of MSNs are more depolarized in Q175 mice as well as show significantly less rheobasic current (the minimum current amplitude that results in depolarization) in HOM mice (Heikkinen et al., 2012), indicating Q175 striatal MSNs become progressively excitable and abnormal corticostriatal neuronal activity emerges as the animals age. Collectively, these studies suggest that dysregulation of MSN firing patterns are a cardinal feature of HD.

Besides abnormal electrophysiological properties of CPN and MSN, dysfunctional network activity has been described both in HD patients and HD models. Neurons operate in a coordinated way and large neuronal population activity can be monitored by local field potentials (LFPs). Spectral analysis of LFPs recorded from the striatum of freely behaving R6/2 mice revealed an increase in power in theta (7–14 Hz) and gamma (35–45 Hz) bands (Miller et al., 2011). Increased power in theta/alpha (4–12 Hz) and low gamma (35–45 Hz) bands also has been observed in LFP activity recorded in the globus pallidus of HD patients (Groiss et al., 2011; Hong et al., 2012). Thus, expression of mutant HTT cause changes in corticostriatal processing that may underlie HD cognitive and behavioral deficits.

Interneurons also may play a role in corticostriatal dysfunction in HD. GABAergic control of striatal MSNs comes primarily from feedforward inhibition derived from local inhibitory interneurons (Tepper et al., 2004). Specifically, parvalbumin-expressing (PV) fast-fast spiking (FS) GABAergic interneurons are the main source of this feedforward inhibition (Gittis et al., 2010). Because PV FS interneurons receive strong cortical innervation (Ramanathan et al., 2002) and respond with faster latency to cortical stimulation than MSNs (Mallet et al., 2005), PV FS cells are able to make feedforward inhibition work effectively. Furthermore, PV FS interneurons project strongly to MSNs (Taverna et al., 2007) with a slight targeting preference for direct-pathway MSNs (Gittis et al., 2010).

In a recent study, significant decreases in striatal PV FS interneurons were observed in postmortem tissue of HD patients with varying degrees of atrophy (Reiner et al., 2013). These changes in interneurons could result in disrupted direct-pathway MSN communication to the GPi. In HD, therefore, local inhibition on the subst P-containing striatonigral D1-enriched MSNs (direct pathway) is limited. According to the highly schematic and simplified circuitry shown in Figure 1, decreased inhibition on the direct pathway would result in more GABA release in GPi, less GABA release in thalamus, and thus more excitation of motor cortex.

Increased firing of FS interneurons has also been observed in HD mouse models. For example, increased GABAergic synaptic activity in symptomatic HD mouse models was observed, primarily from feedforward inhibition of indirect pathway MSNs (Cepeda et al., 2013). Thus, an increase in activity in the indirect pathway from striatum would result in decreased movement during the later stages of HD. In short, inhibitory interneurons act locally in dorsal striatum to influence basal ganglia output.

## Glutamate in Huntington’s Disease

Pioneering studies by Wong et al. (1982) demonstrate perturbation in the synthesis of Glu by corticostriatal neurons. Since then further investigation into Glu receptors and Glu uptake have been investigated in relation to deviations in the corticostriatal pathway.

One major hypothesis is that excitotoxicity underlies striatal neurodegeneration in HD (DiFiglia, 1990). Excitotoxicity can be a result from: (1) an increase in responsiveness of Glu receptors; or (2) an increase in synaptic Glu. The responsiveness of Glu receptors can change due to either an increase in the number of receptors or receptor density or a change in receptor composition or signaling properties. An increase in synaptic Glu could be due to an increase in release or a decrease in uptake. The literature primarily focuses on N-methyl-D-aspartate (NMDA) receptors and removal of excess Glu via glutamate transporter 1 (GLT1).

### NMDA Receptors in HD

NMDA receptors are ionotropic Glu receptors that serve as essential mediators of neuronal function, synaptic transmission, synaptic plasticity, and aspects of neural development (Purves et al., 2008; Iversen et al., 2009). In HD degeneration of MSNs occurs, and while the mechanism behind this selective degeneration is not well understood, convergent evidence supports the role for NMDA receptor mediated excitotoxicity (Beal et al., 1986; Ferrante et al., 1993; Cepeda et al., 2007; Fan and Raymond, 2007).

Striatal injections of NMDA receptor agonists in rodent and non-human primates reproduce the pattern of neuronal damage seen in HD (Beal et al., 1986; Ferrante et al., 1993). Furthermore electrophysiological assessment of pre-symptomatic and symptomatic R6/2 mice found larger NMDA currents and NMDA-induced Ca^2+^ influx compared to littermate controls (Cepeda et al., 2001). It was speculated the striatal neuronal subpopulation that displayed the most elevated response to NMDA application corresponded to indirect MSNs. The increase in NMDA response as well as enhancement of intracellular calcium suggests changes in NMDA receptors signaling could result in excitotoxicity and neuronal death.

NMDA exposure to the MSNs cultured cells resulted in a potentiation in apoptosis of YAC72 and YAC128 MSNs compared to the healthy YAC18 control MSNs. Moreover producing a reduction in NMDA receptor mediated current and calcium influx in YAC72 MSNs to levels seen in YAC18 MSNs resulted in a reduction of NMDA receptor-mediated apoptosis (Shehadeh et al., 2006). Thus, controlling NMDA signaling brought the rate of apoptosis in HD mice to a comparable level seen in WT controls.

However NMDA receptor activation has been shown to promote both neuronal cell survival as well as neuronal cell death (Hardingham and Bading, 2010). Synaptic NMDA receptor activity has been shown to reduce mhtt toxicity and act as a neuroprotectant while extrasynaptic NMDA receptors have been associated with promoting cell death (Okamoto et al., 2009; Milnerwood et al., 2010). Okamoto et al. (2009) found synaptic NMDA receptor activity reduces mutant *htt* toxicity by increasing the formation of non-toxic mutant *htt* inclusions by a process involving the up-regulation of protein chaperones thereby rendering neurons more resistant to mhtt-mediated cell death. In contrast, stimulation of extrasynaptic NMDA receptors increased the vulnerability of neurons to cell death by impairing a neuroprotective CREB-PGC-1α cascade. Furthermore treatment with lower doses of memantine, which blocks extrasynaptic but not synaptic NMDA receptors, improves neuropathological and behavioral manifestations of HD (Okamoto et al., 2009). Also pre-symptomatic YAC128 mice treated at a low level dose that preferentially targets extrasynaptic NMDA receptors resulted in reversal of early signaling and motor deficits (Milnerwood et al., 2010). Though at higher doses, when memantine also blocks synaptic NMDA receptors, memantine worsened the manifestation of HD (Okamoto et al., 2009). This perturbation in the balance between synaptic NMDA and extrasynaptic NMDA receptor activity could contribute to excitotoxicity and neuronal dysfunction in HD.

### Glutamate Uptake in HD

Inadequate Glu uptake has been reported for HD patients as well as transgenic mouse models of HD. The removal of Glu from the synaptic cleft is controlled by several transport proteins; including: GLT1, L-glutamate/L-aspartate transporter (GLAST), and excitatory amino-acid carrier 1 (EAAC1). Note GLT1 is also known as in EAAT2 in humans and is primarily located on glial cells. Glu transporters play a vital role in maintaining the level of extracellular Glu by removing Glu from the synapse; dysregulation of this Glu uptake can result in slow Glu clearance prompting Glu spillover and increased receptor activation (Beart and O’Shea, 2007; Estrada-Sánchez et al., 2008). These high concentrations of Glu can result in excitotoxicity, a pathological process in which neuronal cells are damaged or killed due to excessive stimulation. Therefore the functionality of the Glu transporters plays a critical role in preserving the local integrity of excitatory synaptic transmission (Marcaggi and Attwell, 2004).

Astrocytes not only play a role in Glu uptake via Glu transporters, but recent evidence indicates that mutant *htt* can affect astrocytes in a way that increases Glu release and MSN excitability (Lee et al., 2013; Khakh and Sofroniew, 2014; Tong et al., 2014). Calcium and Glu imaging of astrocytes in the full-length BACHD mouse model showed increased release of Glu into extracellular space. Furthermore BACHD mice have increased *de novo* synthesis of Glu (Lee et al., 2013). This ability of mutant htt to increase Glu production and release could further contribute to the excitotoxicity seen in HD. Additionally, a reduction in astrocyte functional proteins, including GLT1and Kir4.1, has been associated with onset of neurological symptoms (Tong et al., 2014). Restoration of Kir4.1 channels through viral vector administration attenuated MSN excitability, improved gait, and increased the lifespan of HD mice (Khakh and Sofroniew, 2014; Tong et al., 2014). For a more thorough review of astrocytes involvement in circuitry and disease models, see Khakh and Sofroniew (2015).

GLT1 is responsible for 90% of extracellular Glu uptake and is either down regulated or dysfunctional in HD mouse models (Estrada-Sánchez and Rebec, 2012). Although the extracellular concentration of Glu is similar in HD mice to that of WT controls (NicNiocaill et al., 2001; Gianfriddo et al., 2004), the length of time Glu remains in the synapse is prolonged in HD models. A microdialysis study found Glu release is larger and takes longer to return to basal levels in the striatum of R6/1 mice (NicNiocaill et al., 2001), indicating a change in Glu uptake. Moreover, Lievens et al. (2001) showed a decrease of GLT1 mRNA in the striatum and cortex of R6/2 mice accompanied by a concomitant decrease in Glu uptake without a change in GLAST and EAAC1. Other studies have confirmed the decrease in GLT1 protein (Estrada-Sánchez et al., 2009, 2010; Sari et al., 2010), but some of these studies also found a decrease GLAST (Estrada-Sánchez et al., 2009, 2010). A study using the CAG140 KI mouse model found functional changes in EAAC1, but the overall protein level of EAAC1 in HD mice was not significantly different from the level in WT controls (Li et al., 2010). Thus, not only is Glu uptake decreased, suggesting a functional change, but expression of the Glu transporter proteins, especially GLT1, is also decreased, indicating an underlying change in the uptake machinery itself. Further discussion of this machinery and the Glu synapse, including the role of astrocytes in Glu regulation, is described elsewhere (Estrada-Sánchez et al., 2015a).

Because the experiments using the R6 mouse models were carried out before the overt neurological phenotype emerged, the down-regulation of GLT1 appears to persist throughout the progression of HD and is a likely contributor to neurodegeneration. Furthermore, stimulation of layer V afferents innervating the striatum evoked excitatory postsynaptic currents (EPSCs) mediated by Glu. The evoked EPSCs are decreased in Q175 mice, indicating dysregulation of Glu transmission in this model (Menalled et al., 2012).

Dysregulation of Glu also has been observed in human postmortem studies. In 1986, Cross et al. (1986) studied the binding of [3H]-aspartic acid to the high-affinity Glu uptake system and found a significant reduction in [3H]-aspartic acid binding in both the caudate nucleus and putamen of HD subjects. A reduction in the transporter GLT1 mRNA was also observed in the caudate and putamen of HD patients, suggesting a loss of Glu transporter (Arzberger et al., 1997). In a recent study, uptake of [3H]-glutamate was reduced by over 50% in the prefrontal cortex of HD patients with no change in GLAST but a decrease in EAAT2 (Hassel et al., 2008), further demonstrating a dysregulation in Glu clearance. Postmortem evaluation of HD patients revealed a grade-dependent decrease in striatal GLT1 expression (Faideau et al., 2010). Increasing evidence implicates deficits in Glu uptake in HD pathology and that this decrease is, at least in part, due to down regulation of the GLT1 transporter.

Manipulation of GLT1 has been tested as a therapeutic target. Acute administration of ceftriaxone, a β-lactam antibiotic known to elevate GLT1 expression (Rothstein et al., 2005), resulted in attenuation of behavioral deficits. Paw clasping and twitching were reduced in HD mice compared to WT control (Miller et al., 2008). These results not only indicated impaired Glu uptake is a major factor underlying HD pathophysiology and symptomology, but restoration of GLT1 could be a viable treatment for HD. Furthermore ascorbic acid, which is associated with GLT1, appears decreased in HD mice. Following treatment with ceftriaxone, ascorbic acid was increased in HD mice (Miller et al., 2012). These results suggest a dysfunction of the Glu system in HD. An increase in Glu uptake via GLT1 is a viable therapeutic strategy because it removes excess Glu from the synapse which can result in excitotoxicity.

## Dopamine in Huntington’s Disease

Aberrant DA signaling underlying behavioral abnormalities in HD was first proposed in 1970 based on evidence that asymptomatic offspring of individuals with HD developed dyskinesia following levodopa (L-DOPA) administration (Klawans et al., 1970). Further studies of HD patients suggest increased DA release induces chorea while a reduction in DA leads to akinesia. Levels of DA were found to be 11-fold higher in the cerebrospinal fluid (CSF) of early-stage HD patients compared to healthy individuals (Garrett and Soares-da-Silva, 1992), but decreased DA was found in patients studied at a later stage in the disease (Kish et al., 1987). In line with this finding, recent work has shown biphasic changes in DA release in the R6/1 mouse model of HD: enhanced DA release during pre-symptomatic stages and severely attenuated DA release in symptomatic animals (Dallérac et al., 2015). Collectively, these results suggest a biphasic, age-dependent change in DA transmission in HD.

The progressive reduction in striatal DA levels during later stages of HD has been confirmed in multiple studies (Hickey et al., 2002; Johnson et al., 2006; Ortiz et al., 2010, 2011; Callahan and Abercrombie, 2011; Dallérac et al., 2015), but it remains unclear if early stages promote DA excess. One study using transgenic HD rats showed increased striatal tyrosine hydroxylase expression and increased DA neurons during the early symptomatic stage (Jahanshahi et al., 2010). These rats, however, also have been shown to have impaired DA release (Ortiz et al., 2012). Further work is required to clarify DA changes during the hyperkinetic stages of HD.

Deficits in DA levels and/or release during late HD stages in rodent models have been attributed either to impaired vesicle loading (Suzuki et al., 2001) or to a reduction in the DA readily releasable pool (Ortiz et al., 2010). *In vitro* studies of R6/2 and R6/1 mice have shown a decrease in extracellular DA concentrations as well as a decrease in DA release (Johnson et al., 2006) and uptake (Ortiz et al., 2011). The decrease in DA release emerged during symptom onset and became significant as the animals aged. Importantly, both R6/2 and R6/1 mice have late-stage reductions in DA release, but only R6/1 mice have decreased uptake. Moreover, the decrease in uptake occurred towards the end of the R6/1 lifespan, suggesting that the R6/2-R6/1 uptake difference could be explained by the increased lifespan of the R6/1 model (Mangiarini et al., 1996).

In both HD patients and mouse models, D1 and D2 receptors are compromised during the early as well as late stages of the disease (Chen et al., 2013). An autoradiography study found a decrease in both DA receptor families (Richfield et al., 1991). Positron emission tomography studies have also found decreased striatal D1 and D2 receptors in asymptomatic carriers of mutant *htt* (Weeks et al., 1996; van Oostrom et al., 2009). Multiple studies using the R6 line have also found decreased D1 and D2 receptors signaling and receptor binding (Cha et al., 1998; Bibb et al., 2000; Ariano et al., 2002). A significant reduction in D1 and D2 mRNA was also found in YAC128 mice (Pouladi et al., 2012). Collectively these studies indicate DA receptors, which are involved in modulating basal ganglia circuitry, are disrupted throughout HD progression and in some cases prior to disease onset.

The traditional, albeit simplified view of basal ganglia circuitry, proposed behavioral abnormalities in HD are the result of initial dysregulation of the striatopallidal MSNs of the D2-enriched indirect pathway, resulting in hyperkinetic choreic movements. This is then followed by dysregulation of the striatonigral D1-enriched MSNs of the direct pathways, resulting in hypokinesia (Spektor et al., 2002). However recent studies have suggested a counter view, the direct pathway is affected first by D1 receptor activation. Striatal MSNs receive glutamatergic inputs modulated by DA (Cepeda and Levine, 2006). Different DA receptors are localize in different striatal cell populations with less than 10% of D1 and D2 MSNs co-localized (Deng et al., 2006; Lobo et al., 2006; Cepeda et al., 2008). This lead to the idea that DA modulates the direct and indirect pathway by opposing action, with DA release increasing activity in the direct pathway and reducing activity in the indirect pathway (see above; DeLong and Wichmann, 2007).

However it has been difficult to parcel out the exact mechanism by which D1 and D2 DA receptors influence the direct and indirect pathway. Recently animal expressing enhanced green fluorescent protein (GFP) in either D1 or D2 receptors have been studied. Using electrophysiology with these mice a DA receptor-specific modulation of EPSC and NMDA and AMPA currents was found. D1 receptor agonist increased EPSC in D1 MSNs projecting to the direct pathway whereas D2 receptor agonist decreased EPSCs in D2 cells of the indirect pathway. Changes in NMDA and AMPA current were also DA receptor-specific. A D1 agonist resulted in increased current in direct pathway MSNs, and a D2 receptor agonist resulted in decreased NMDA and AMPA current in the indirect pathway MSNs (André et al., 2010). Thus Glu receptor-mediated responses in MSNs are modulated by the type of DA receptor abundantly expressed in that cell.

Additionally, these results suggest D1 receptor activation leads to increased Glu receptor response on MSNs in the direct pathway, which would result in increased GABA release to the GPi resulting in hyperkinetic movement. An electrophysiological study testing full-length HD mouse models crossed with mice expressing enhanced GFP found direct pathway MSNs received more excitatory inputs than control animals during the early hyperkinetic stage. The indirect pathway MSNs were not as affected during this early stage, and during the hypokinetic stage both pathways received less excitatory inputs compared to WT controls (André et al., 2011; Galvan et al., 2012). Further suggesting increased DA in the early stages of HD contributes to the hyperkinetic symptoms of HD by modifying the direct pathway first.

Morphological assessments have found the indirect pathway to be affected initially in HD (see above). Electrophysiological data from mice expressing GFP now suggest the direct pathway is affected in the early stages of HD via D1 receptor activation. More than likely it is a combination of D1 receptor activation in the direct pathway in conjunction with decreased function of the indirect pathway. Since both theories would result in increased GABAergic output to the direct pathway, the ultimate result is an exacerbated HD phenotype.

## Conclusion

HD is a multifaceted disorder displaying cognitive, psychiatric, and motor deficits. The dysregulation in the corticostriatal pathway contributes to these associated characteristics of HD and potentially underlie the HD phenotype. Dysregulation of Glu and DA have been documented in HD patients as well as rodent models, and the abnormal electrophysiology, due to these neurotransmitters, further indicates that disrupted communication in the corticostriatal pathway in HD.

## Author Contributions

Both authors contributed to this review article. KDB: prepared a draft, which was updated and edited by GVR.

## Funding

We gratefully acknowledge support from the CHDI Foundation (Grants A-7449).

## Conflict of Interest Statement

The authors declare that the research was conducted in the absence of any commercial or financial relationships that could be construed as a potential conflict of interest.
